# Prevalence and Antimicrobial Susceptibility Profile of* Salmonella* Serovars Isolated from Slaughtered Cattle in Addis Ababa, Ethiopia

**DOI:** 10.1155/2018/9794869

**Published:** 2018-11-04

**Authors:** Lidya Ketema, Zerihun Ketema, Bitsu Kiflu, Haile Alemayehu, Yitagele Terefe, Mohammed Ibrahim, Tadesse Eguale

**Affiliations:** ^1^Beshir Husein Veterinary Drugs and Equipment Wholesale, P.O. Box 181979, Addis Ababa, Ethiopia; ^2^Sebeta Municipal Abattoir, Sebeta, Ethiopia; ^3^Ministry of Agriculture, P.O. Box 170042, Addis Ababa, Ethiopia; ^4^Aklilu Lemma Institute of Pathobiology, Addis Ababa University, P.O. Box 1176, Addis Ababa, Ethiopia; ^5^College of Veterinary Medicine, Haramaya University, P.O. Box 138, Dire Dawa, Ethiopia; ^6^Jigjiga University, P.O.Box 1020, Jigjiga, Ethiopia

## Abstract

*Salmonella *is one of the top causes of foodborne bacterial illnesses in humans. The primary sources of human* Salmonella* infection are food producing animals such as cattle, poultry, and swine. A cross-sectional study was undertaken to estimate the prevalence and to determine the serovar distribution and antimicrobial susceptibility profiles of* Salmonella* spp. isolated from fecal (n=567) and carcass swab (n=159) samples of slaughtered cattle at Addis Ababa Abattoir Enterprise and Kara'alo PLC, Abattoirs, in Addis Ababa, Ethiopia between January 2014 and April 2015.* Salmonella* isolation was conducted according to Global Foodborne Infections Network Laboratory Protocol and isolates were confirmed by genus specific PCR and serotyped by slide agglutination test. Susceptibility of the isolates to 17 antimicrobials was testedusing the Kirby-Bauer disk diffusion method according to the guidelines of the Clinical and Laboratory Standards Institute. Out of the total 726 samples examined, 27 (3.7%) were positive for* Salmonella. Salmonella *was detected in 4.1% (23/567) fecal and 2.5% (4/159) carcass swab samples. Twelve different serovars were identified and the most predominant serovars were* S*. Dublin (n=10, 35.7%) and* S*. Virchow (n=5, 17.9%), followed by* S*. Braendrerup,* S*. Haifa, and* S*. Saintpaul which were isolated from 2 samples each (7.1%). All of the* Salmonella* isolates investigated were resistant or intermediately resistant to four or more of the 17 drugs tested. High resistance rate was recorded to streptomycin 25 (89.3%), cephalothin 20 (71.4%), ampicillin 19 (67.9%), and amoxicillin+clavulanic acid 19 (67.9%). Resistance to five or more antimicrobials was detected in 20 (71.5%) of the isolates. Multidrug resistance to more than 7 antimicrobials was detected in 5 (17.9%) of the isolates. Isolation of such multidrug resistant strains of* Salmonella *from slaughtered cattle poses a major public health concern. These findings imply the need for a strict biosecurity and regulation of antimicrobial use across the country.

## 1. Introduction

*Salmonella* is one of the most important foodborne bacteria in the world and infection with* Salmonella* spp. is a major cause of diarrhea in children and adults [1].* Salmonella *belongs to the* Enterobacteriaceae *family which also includes pathogens such as* Escherichia coli, Shigella, *and* Klebsiella *[2]. Members of the genus* Salmonella *are ubiquitous pathogens that infect a wide variety of mammals, birds, fish, and reptiles, as well as humans. There are 2 species of* Salmonella: Salmonella enterica and Salmonella bongori*.* Salmonella enterica* is classified into 6 subspecies of which* Salmonella enterica* subspecies enterica is the dominant subspecies affecting humans and domestic animals [3]. There are currently over 2,700* Salmonella *serovars [4], which are serologically identified by antigenic variation in the O (Lipopolysaccharide), H (Flagella), and Vi (Capsular) antigens in accordance with the Kauffmann–White scheme [4, 5].

Salmonellosis represents an important public health problem among the common bacterial foodborne pathogens worldwide. Estimated global burden of 93.8 million gastroenteritis cases and 155,000 deaths are due to* Salmonella* species annually, of which 85.6% is foodborne [6]. Human salmonellosis has been associated with contaminated food products, mainly those of animal origin such as poultry, beef, pork, and dairy products, as well as direct contact with infected animals [7–9].

The presence of* Salmonella* in food animals and the consequent cross-contamination of edible carcass present a significant food-safety hazard [10]. Food animals such as cattle may carry* Salmonella* at slaughter and can serve as sources of contamination and provide an opportunity for entry of the pathogen into the food products. This implies that the presence of* Salmonella* in slaughter cattle and slaughter house environment and the potential cross-contamination of carcasses and edible organs can pose a significant food-safety hazards [3]. There is little available data on* Salmonella* presence in cattle feces and carcass swab and such information is important to understand the impact of the bacteria on food producing animals and the subsequent risk to humans which consume cattle products. The aim of the current study was therefore to estimate* Salmonella *prevalence in feces and carcass swab of cattle slaughtered at Addis Ababa Abattoir Enterprise and Kara'alo PLC abattoir. Furthermore, the isolates were serotyped and tested for antimicrobial susceptibility.

## 2. Materials and Methods

### 2.1. Study Area and Study Design

The study was conducted in Addis Ababa between January 2014 and April 2015. Addis Ababa is the capital city and administration center for the Federal Democratic Republic of Ethiopia. There are four big abattoirs in Addis Ababa town, of which three of them are government owned and the other one is a private limited company (PLC). Of these four abattoirs, we selected two abattoirs, namely, Addis Ababa Abattoir Enterprise and Kara'alo PLC Abattoir.

Addis Ababa Abattoir is the largest government owned abattoir in Addis Ababa, established with the objective of providing wholesome and hygienically slaughtered meat to the public. The enterprise gives slaughtering service for average of 1200 cattle, 1000 sheep, and goats and 10 camels per day. Kara'alo PLC abattoir is the only private abattoir found in Addis Ababa which is located in Kara'alo area, Addis Ababa. The abattoir gives slaughtering services for the average of 350 cattle per day. The animals slaughtered in these abattoirs originate from different parts of the country.

*Study Design and Sample Size Determination. *A cross-sectional study was conducted on apparently healthy cattle that were slaughtered at both abattoirs. The required sample size of the study was determined by the formula given by Thrusfield [11] with 95% confidence interval and 5% desired precision. Based on the mean prevalence report of 10.6% from previous work [12], we calculated the samples size as follows.

N = (1.96)^2^x Pexp (1- Pexp)/d^2^, where N is required sample size, p is expected prevalence, and d is desired absolute precision of 0.05. Therefore, the calculated sample size was 146, but, to increase the precision of the study and to increase the number of* Salmonella* isolates, a total of 720 samples (567 fecal and 159 Swab samples) were collected from the two abattoirs.

### 2.2. Sample Collection

Fresh fecal sample was collected directly from the rectum of each animal during antemortem inspection before slaughter using disposable gloves. Fecal samples were collected into sterile zippered plastic bags directly from rectum using disposable gloves and transported to Microbiology Laboratory, Aklilu Lemma Institute of Pathobiology, Addis Ababa University, in ice box. Similarly, carcass swab was sampled by rubbing the entire surface of each carcass (both sides) once from the hind quarter to the forequarter, uniformly using sterile cotton swab. Each swab sample was then placed into screw caped test tubes containing 10 ml of sterilized buffered peptone water (BPW) (Becton Dickinson, Sparks, MD), placed in an ice box with an ice pack and transported to Microbiology Laboratory, Aklilu Lemma Institute of Pathobiology, within 3–4 h of collection.

### 2.3. Bacterial Isolation and Identification

Isolation and identification of* Salmonella* were conducted using conventional methods [13]. Briefly, 10 g of feces was preenriched in 90 ml of sterile buffered peptone water (BPW) (Becton Dickinson, Sparks, MD**)** and incubated overnight at 37°C. Similarly, the carcass swab sample in BPW was placed in incubator overnight at 37°C. A 100*μ*l of each preenriched suspension was added into 9.9 ml of Rappaport-Vassiliadis enrichment Broth (RVB) (Oxoid, USA) and incubated at 42°C for 24 h. At the same time, 1 ml of the suspension was also transferred to 10 ml of Tetrathionate broth (TTB) (Oxoid, USA) and incubated for 24 h at 37°C. It was then streaked from both RVB and TTB to Xylose Lysine Tergitol 4 (XLT-4) (Oxoid, USA) selective media and the plates were incubated at 37°C for 24 to 48 h. Presumptive* Salmonella *colonies were further investigated biochemically using Triple Sugar Iron agar, Urea, Citrate, and Lysine Iron Agar slants. Those colonies with typical* Salmonella *biochemical properties were then further confirmed by genus specific PCR [14]. A reference strain of* S*. Typhimurium (ATCC 14028) was used as a positive control during biochemical analysis and PCR. One confirmed* Salmonella* isolate from each positive sample was stored at −80°C in 20 % glycerol until further testing. When* Salmonella* was recovered from samples enriched with both RV and TTB, they were considered as different strains until we conduct antimicrobial susceptibility test. Isolates with different antimicrobial susceptibility profile were considered different strains and both of them were submitted for serotyping. However, when isolates exhibited identical antimicrobial susceptibility profile, they were considered as the same strain and only one isolate was randomly selected for further investigation.

### 2.4. *Salmonella* Serotyping and Phage Typing

*Salmonella* isolates were serotyped and phage-typed at the Public Health Agency of Canada, World Organization for Animal Health (OIÉ) Reference Laboratory for Salmonellosis, Guelph, Ontario, Canada, as described previously [15].

### 2.5. Antimicrobial Susceptibility Testing

Susceptibility of the isolates to 17 antimicrobials was determined using the disk diffusion method according to the guidelines of the Clinical and Laboratory Standards Institute [16]. The following antimicrobials (Sensi-Discs, Becton, Dickinson and Company, USA) and disc potencies (*μ*g) were used: amikacin (30*μ*g), ampicillin (10 *μ*g), amoxicillin-clavulanic acid 20/10 (30 *μ*g), chloramphenicol (30*μ*g), ceftriaxone (30*μ*g), cephalothin (30 *μ*g), ciprofloxacin (5*μ*g), cefoxitin (30 *μ*g), gentamycin (10*μ*g), kanamycin (30*μ*g), sulfamethoxazole+trimethoprim (23.75 *μ*g/1.25 *μ*g), trimethoprim (5 *μ*g), tetracycline (30*μ*g), sulfisoxazole (1000 *μ*g), streptomycin (10 *μ*g), nitrofurantoin (30*μ*g), and nalidixic acid (30*μ*g), and the interpretation of the categories of susceptible, intermediate, or resistant was done based on the CLSI guidelines [16]. Reference strain of* Escherichia coli* ATCC 25922 was used as a quality control.

### 2.6. Data Analysis

Data was generated from combination of records from data collection and laboratory results. All the data and corresponding laboratory results were entered into Microsoft Excel, edited, coded, and analyzed using SPSS version 15.0. Prevalence of* Salmonella* was calculated as a percentage of* Salmonella* culture-positive samples among the total number of samples examined.

## 3. Results

### 3.1. Prevalence of* Salmonella*

*Salmonella* was isolated from 27 of the 726 samples examined resulting in an overall* Salmonella* prevalence of 3.7%. From the total of 567 fecal samples, 23 (4.1%) and from 159 carcass swab samples 4 (2.5%) were found positive for* Salmonella. *Summary of the prevalence of* Salmonella *from fecal samples and carcass swab samples in the two abattoirs is presented in Table 1.

### 3.2. *Salmonella *Serovar Distribution

A total of 12 different* Salmonella *serovars were identified and the most predominant serovars identified were* S*. Dublin 10 (35.7%) and* S*. Virchow 5 (17.86%). Other serovars like* S*. Braendrerup,* S*. Haifa, and* S*. Saintpaul were also isolated from 2 samples each (7.14%). Two serovars were isolated from single sample, namely,* S*. Haifa and* S*. Kottbus: one from fecal sample obtained from a cattle slaughtered in Addis Ababa Abattoir enriched with RV and the other from the same sample enriched with TTB.  Table 2 shows the distribution of* Salmonella *serovars and their antigenic formula.

### 3.3. Antimicrobial Susceptibility of* Salmonella* Isolates

The antimicrobial susceptibility profile of* Salmonella* isolates is shown in Table 3. High resistance rate was recorded among isolates to streptomycin 25 (89.3%), cephalothin 20 (71.4%), ampicillin 19 (67.9%), and amoicillin+clavulanic acid 19 (67.9%). All isolates were susceptible to ceftriaxone and chloramphenicol. All* S*. Dublin isolates were resistant to ampicillin, amoxicillin+clavulanic acid, cephalothin, and streptomycin. All of the* Salmonella* isolates investigated were resistant or intermediately resistant to 4 or more of the 17 drugs tested. Resistance or intermediate resistance to five or more drugs was detected in 20 (71.5%) of the isolates and resistance to more than 7 antimicrobials was detected in 5 (17.9%) of the isolates. A single* S*. Kentucky isolated from Addis Ababa Abattoir was resistant to 11 of the 17 antimicrobials tested (Table 4).

## 4. Discussion

Foodborne gastroenteritis caused by non-typhoidal* Salmonella *represents a major public health problem worldwide. As* Salmonella *is transmitted through contaminated food or water, its presence in food animals and food animal products has relevant public health implications. Thus, monitoring food safety is a key point in preventing and controlling the spread of* Salmonella*, as well as in providing healthier food product [17].

In the current study, overall prevalence of* Salmonella *was 3.7% (fecal= 4.1% and carcass swab 2.5%), which is a little bit lower than that reported from Addis Ababa Abattoir [18] and Debrezeit slaughter house [19] which reported pooled prevalence to be 10.6% and 7.1%, respectively. A much higher prevalence of 14% was also reported from beef cattle in Ethiopia [20]. The prevalence from carcass swab (2.5%) in the current study is in line with the 2% prevalence reported earlier [20]. However, higher rate of recovery of* Salmonella *(4.8%) was reported from carcass swab [21] in slaughtered cattle at Bahir Dar town, North Ethiopia. The probable reason for the fecal and carcass swab prevalence disparity from other studies could be due to seasonal variation in* Salmonella* shedding among animals, difference in infection prevention practices by animal owners, and other factors such as variation in hygiene status of the slaughter houses and difference in the* Salmonella* isolation protocol employed in each study.

In our study, we found* S*. Dublin to be the predominant serovar followed by* S*. Virchow. Similarly, previous studies [12, 18, 19] reported* S*. Dublin as the most predominate serovar in slaughtered cattle and beef in Ethiopia. A single I:ROUGH-O:g,p:-isolate obtained from fecal sample of cattle is also likely atypical strain of* S*. Dublin as its antimicrobial susceptibility profile is closely related to most of the* S*. Dublin strains in this study. Unlike our study,* S*. Virchow was not the common serovar isolated from cattle and carcass sample in the previous studies. However, recent study reported* S*. Virchow to be one of the dominant serovars circulating in dairy cattle in Addis Ababa [15].

In the current study, resistance to 4 or more antimicrobials was observed in the isolates which is much higher than the reports by previous studies [20, 22] in* Salmonella *serovars isolated from beef and other food of animal origin. Other studies in Ethiopia also reported a much lower rate of resistance to common antimicrobials used in both animal and human health [19, 21]. This difference may be due to the increasing rate of inappropriate utilization of antimicrobials in cattle farms which favored selection for resistant bacterial strains.

Higher resistance rate observed to streptomycin could be due to the fact that it is among the most commonly used antimicrobials for treatment of various infectious diseases of livestock. Similarly, the occurrence of high rate of resistance to ampicillin and amoxicillin+clavulanic acid among isolates in this study could partly be due to coselection of resistance phenomenon with penicillin, a drug frequently prescribed alone or in combination with streptomycin in veterinary practice in Ethiopia [23]. All isolates in the current study showed multidrug resistance to more than four drugs which is alarming for both human and animal health sector. This observation indicates the potential importance of cattle as a source of antimicrobial resistant* Salmonella *serovars to commonly used antimicrobials including ampicillin, amoxicillin+clavulanic acid, streptomycin, and tetracycline. The fact that all isolates in the current study were susceptible to ceftriaxone and chloramphenicol might be due to low use of these two drugs in veterinary medicine in the country.

## 5. Conclusion

Antimicrobial resistant bacteria of animal origin are considered an important contributor to the overall phenomenon of increased antimicrobial resistance in bacterial pathogen of public health importance. Use of antimicrobials in one or the other way contributes to the emergence and spread of antimicrobial resistance. Therefore, judicious use of antimicrobials in beef cattle and other food animals is recommended to delay the emergence and spread of antimicrobial resistant bacteria and resistance genetic markers.

## Figures and Tables

**Table 1:** Summary of the prevalence of *Salmonella *and sample types.

**Abattoir Name**	**Sample type**	**Numbers examined**	**No. positive (**%**)**
Addis Ababa	Fecal	282	11 (3.9%)
	Swab	60	2 (3.3%)

Subtotal		342	13

Kara'alo	Fecal	285	12 (4.2%)
	Swab	99	2 (2.0%)

Subtotal		**384**	**14**

**Total**		**726**	**27 (3.7**%**)**

**Table 2:** *Salmonella *serovars isolated from fecal and swab samples of cattle slaughtered at Addis Ababa, Ethiopia.

Serovars	Antigenic formula	No. isolated from each Abattoirs		Total (%)
		Addis Ababa	Kara'alo	
Dublin	9,12:g,p:-	0	10	10 (35.7)
Virchow	6,7:r:1,2	2	3	5 (17.9)
Braendrerup	6,7:e,h:e,n,z15	2	0	2 (7.1)
Saintpaul	4:e,h:1,2	2	0	2 (7.1)
Haifa	4:z10:1,2	2	0	2 (7.1)
Kottbus	6,8:e,h:1,5	1	0	1 (3.6)
Kentucky	8,20:i:z6	1	0	1 (3.6)
Mikawasima	6,7:y:e,n,z15	1	0	1 (3.6)
Typhimurium phage type 3	4,5:i:1,2	1	0	1 (3.6)
Typhimurium phage type 193	4,5:i:1,2	1	0	1 (3.6)
Typhimurium phage type 4	4,5:i:1,2	1	0	1 (3.6)
I:ROUGH-O:g,p:-	-:g,p:-	0	1	1 (3.6)

**Total**				**28 (100)**

**Table 3:** *Salmonella *serovar distribution and frequency of resistance to various antimicrobials.

Antimicrobials tested	*Salmonella *serovars and No. (%) of isolates *∗*resistant to various antimicrobials (n=28)										
	Dublin	Virchow	Typhimurium	Saintpaul	Braenderup	Haifa	Kottbus	Kentucky	Mikawasima	I:ROUGH-O:g,p:-	No. (%) resistant
	n=10	n=5	n=3	n=2	n=2	n=2	n=1	n=1	n=1	n=1	
Amp	10 (100)	3 (60)	2 (66.7)	2 (100)	-	-	-	1 (100)	-	1 (100)	19 (67.9)
Amc	10 (100)	3 (60)	2 (66.7)	2 (100)	-	-	-	1 (100)	-	1 (100)	19 (67.9)
Cf	10 (100)	3 (60)	2 (66.7)	2 (100)	-	-	1 (100)	1 (100)	-	1 (100)	20 (71.4)
Cro	-	-	-		-	-	-		-	-	0
Fox	1 (10)	-	-		-	-	-		-	1 (100)	2 (7.1)
An	-	1 (20)	-	-	1 (50)	1 (50)	-	-	1 (100)	-	4 (14.3)
Gm	1 (10)		-	-	-	-	-	1 (100)	-	-	2 (7.1)
K	3 (30)	5 (100)	2 (66.7)	1 (50)	1 (50)	2 (100)	1 (100)	1 (100)	-	-	16 (57.1)
S	10 (100)	5 (100)	2 (66.7)	2 (100)	2 (100)	1 (50)	1 (100)	1 (100)	1 (100)	-	25 (89.3)
Sxt	-	-	1 (33.3)	-	-	-	-	-	-	-	1 (3.6)
Cip	-	1 (20)	2 (66.7)	2 (100)	2 (100)	1 (50)	-	1 (100)	1 (100)	-	10 (35.7)
Na	1 (10)	-	-	-	-	-	-	1 (100)		-	2 (7.1)
Te	-	2 (40)	1 (33.3)	2 (100)	2 (100)	1 (50)	1 (100)	1 (100)	1 (100)	-	11 (39.3)
C	-	-	-	-	-	-	-	-	-	-	0
Tmp	1 (10)	-	1 (33.3)	-	-	-	-	-	-	-	2 (7.1)
Su	1 (10)	2 (40)	-	2 (100)	2 (100)	2 (100)	1 (100)	1 (100)	1 (100)	-	12 (42.9)
Nitro	-	2 (40)	2 (66.7)	-	1 (50)	2 (100)	1 (100)	1 (100)	1 (100)	-	10 (35.7)

An: amikacin, Amp: ampicillin, Amc: amoxicillin+clavulanic acid, C: chloramphenicol, Cro: ceftrioxone, Cf: cephalothin, Cip: ciprofloxacin, Fox: cefoxitin, Gm: gentamicin, K: kanamycin, Sxt: sulfamethoxazole+trimethoprim, Tmp: trimethoprim, Te: tetracycline, Suv: sulfisoxazole, S: streptomycin, Nitro: nitrofurantoin, and Na: nalidixic acid. *∗*Isolates intermediately resistant were also considered resistant.

**Table 4:** Antimicrobial resistance pattern of *Salmonella *serovars isolated from slaughtered cattle.

Serovar	**No.**	Resistance Pattern	
		Intermediate	Resistant
Braendrerup	1	Cip,Te,Su,S	-
Braendrerup	1	An,Cip,K,Te,Su,S	Nitro
Dublin	2	K	Amp,Amc,Cf,S
Dublin	2	S	Amp,Amc,Cf
Dublin	1	S	Amp,Amc,Cf,Gm
Dublin	1	S,Su,Tmp	Amp,Amc,Cf
Dublin	1	S	Amp,Amc,Cf
Dublin	1	-	Amp,Amc,Cf,S
Dublin	1	Fox,K	Amp,Amc,Cf,S
Dublin	1	Na,S	Amp,Amc,Cf
Haifa	1	K,Nitro	Te,Su
Haifa	1	An,Cip,K,Su,S,Nitro	-
Kottbus	1	Cf,K,S	Te,Su,Nitro
Kentucky	1	K,Nitro	Amp,Amc,Cf,Cip,Gm,Te,Su,S,Na
Mikawasima	1	An,Cip,Te	Su,S,Nitro
Saintpaul	1	Amc,Cip,K,Su,S	Amp,Cf, Te
Saintpaul	1	Amp,Amc,Cf,Cip,Su,S	Amp,Cf,Te
Typhimurium phage type 3	1	Cip,K,Nitro,S	Amp,Amc,Cf
Typhimurium phage type 93	1	-	Sxt,Tmp,Te,S
Typhimurium phage type 4	1	Cip,K,Nitro	Amp,Amc,Cf
Virchow	1	K,Te,Su,S	Nitro
Virchow	1	Cip,K,Te,Su,S,Nitro	-
Virchow	1	An, K	Amp,Amc,Cf,S
Virchow	2	K,S	Amp,Amc,Cf
I:ROUGH-O:g,p:-	1	Fox	Amp,Amc,Cf

An, amikacin; Amp, ampicillin; Amc, amoxicillin and clavulanic acid; Cf, cephalothin; C, chloramphenicol; Cro, ceftriaxone; Cip, ciprofloxacin; Fox, cefoxitin; Gm, gentamicin; K, kanamycin; Tmp, trimethoprim; Sxt, sulfamethoxazole + trimethoprim; Te, tetracycline; Su, sulfisoxazole; S, streptomycin; Nitro, nitrofurantoin; Na, nalidixic acid, N, neomycin.

## Data Availability

The data used to support the findings of this study are included within the article.

## References

[1] Al-Abri S. S., Beeching N. J., Nye F. J. (2005). Traveller's diarrhoea. *The Lancet Infectious Diseases*.

[2] Ellermeier C. D., Slauch J. M., Dworkin M., Falkow S., Rosenberg E., Schleifer K., Stackebrandt E. (2006). The Genus Salmonella. *he Prokaryotes: Volume 6: Proteobacteria: Gamma Subclass*.

[3] Uzzau S., Brown D. J., Wallis T. (2000). Host adapted serotypes of *Salmonella enterica*. *Epidemiology and Infection*.

[4] Guibourdenche M., Roggentin P., Mikoleit M. (2010). Supplement 2003–2007 (No. 47) to the White-Kauffmann-Le Minor scheme. *Research in Microbiology*.

[5] Grimont P. A. D., Weill F. X. (2007). *Antigenic formulae of the Salmonella Serovars*.

[6] Majowicz S. E., Musto J., Scallan E. (2010). The global burden of nontyphoidal *Salmonella gastroenteritis*. *Clinical Infectious Diseases*.

[7] Bouchrif B., Le Hello S., Pardos M. (2009). Ceftazidime-resistant Salmonella enterica, Morocco. *Emerging Infectious Diseases*.

[8] Fey P. D., Safranek T. J., Rupp M. E. (2000). Ceftriaxone-resistant Salmonella infection acquired by a child from cattle. *The New England Journal of Medicine*.

[9] Bartholomew M. L., Heffernan R. T., Wright J. G. (2014). Multistate outbreak of salmonella enterica serotype enteritidis infection associated with pet guinea pigs. *Vector-Borne and Zoonotic Diseases*.

[10] Kikuvi G. M., Ombui J. N., Mitema E. S. (2010). Serotypes and antimicrobial resistance profiles of Salmonella isolates from pigs at slaughter in Kenya. *The Journal of Infection in Developing Countries*.

[11] Thrusfield M. (2007). *Veterinary Epidemiology*.

[12] Nyeleti C., Hildebrandt G., Kleer J., Molla B. (2000). Prevalence of *Salmonella* in Ethiopian cattle and minced beef. *Berlin und Münchener Tierärztliche Wochenschrift*.

[13] WHO (2010). WHO Global Foodborne Infections Network Laboratory Protocol. *Isolation of Salmonella spp. From Food and Animal Feace*.

[14] Cohen N. D., Neibergs H. L., McGruder E. D. (1993). Genus-specific detection of salmonellae using the polymerase chain reaction (PCR). *Journal of Veterinary Diagnostic Investigation*.

[15] Eguale T., Engidawork E., Gebreyes W. A. (2016). Fecal prevalence, serotype distribution and antimicrobial resistance of *Salmonellae* in dairy cattle in central Ethiopia. *BMC Microbiology*.

[16] CLSI (2013). *Performance standards for antimicrobial susceptibility testing; twenty-third informational SupplementM100-S23*.

[17] Bouchrif B., Paglietti B., Murgia M. (2009). Prevalence and antibiotic-resistance of *Salmonella* isolated from food in Morocco. *The Journal of Infection in Developing Countries*.

[18] Nyeleti C., Molla B., Hildebrandt G., Kleer J. (2000). The prevalence and distribution of Salmonellae in slaughter cattle, slaughterhouse personnel and minced beef in. *Bulletin of Animal Health and Production in Africa*.

[19] Alemayehu D., Molla B., Muckle A. (2003). Prevalence and antimicrobial resistance pattern of *Salmonella* isolates from apparently healthy slaughtered cattle in Ethiopia. *Tropical Animal Health and Production*.

[20] Sibhat B., Molla Zewde B., Zerihun A. (2011). Salmonella Serovars and Antimicrobial Resistance Profiles in Beef Cattle, Slaughterhouse Personnel and Slaughterhouse Environment in Ethiopia. *Zoonoses and Public Health*.

[21] Alemu S., Zewde B. M. (2012). Prevalence and antimicrobial resistance profiles of *Salmonella enterica* serovars isolated from slaughtered cattle in Bahir Dar, Ethiopia. *Tropical Animal Health and Production*.

[22] Zewdu E., Cornelius P. (2009). Antimicrobial resistance pattern of *Salmonella* serotypes isolated from food items and personnel in Addis Ababa, Ethiopia. *Tropical Animal Health and Production*.

[23] Tadesse G. (2015). A meta-analysis of the proportion of animal *Salmonella* isolates resistant to drugs used against human salmonellosis in Ethiopia. *BMC Infectious Diseases*.

